# Quantification of Error Sources with Inertial Measurement Units in Sports

**DOI:** 10.3390/s22249765

**Published:** 2022-12-13

**Authors:** Haye Kamstra, Erik Wilmes, Frans C. T. van der Helm

**Affiliations:** 1 Biomechanical Engineering, Faculty of Mechanical, Maritime and Materials Engineering, Delft University of Technology, 2628 CN Delft, The Netherlands; 2 Amsterdam Movement Sciences, Department of Human Movement Sciences, Faculty of Behavioural and Movement Sciences, Vrije Universiteit Amsterdam, 1081 BT Amsterdam, The Netherlands; 3 FIFA Medical Centre of Excellence, Royal Netherlands Football Association, 3707 HX Zeist, The Netherlands

**Keywords:** inertial measurement unit, soft tissue artefact, orientation filter, error quantification, football

## Abstract

Background: Inertial measurement units (IMUs) offer the possibility to capture the lower body motions of players of outdoor team sports. However, various sources of error are present when using IMUs: the definition of the body frames, the soft tissue artefact (STA) and the orientation filter. Methods to minimize these errors are currently being used without knowing their exact influence on the various sources of errors. The goal of this study was to present a method to quantify each of the sources of error of an IMU separately. Methods: An optoelectronic system was used as a gold standard. Rigid marker clusters (RMCs) were designed to construct a rigid connection between the IMU and four markers. This allowed for the separate quantification of each of the sources of error. Ten subjects performed nine different football-specific movements, varying both in the type of movement, and in movement intensity. Results: The error of the definition of the body frames (11.3–18.7 deg RMSD), the STA (3.8–9.1 deg RMSD) and the error of the orientation filter (3.0–12.7 deg RMSD) were all quantified separately for each body segment. Conclusions: The error sources of IMU-based motion analysis were quantified separately. This allows future studies to quantify and optimize the effects of error reduction techniques.

## 1. Introduction

Human motion analyses can provide valuable information regarding athletic performance in sports. Currently the gold standard for human motion analysis is an optoelectronic system [1], it uses multiple cameras and skin placed markers on bony anatomical landmarks to capture the motion of the human body. However, this method has some important limitations, including a restricted measurement volume, extensive set-up procedures, high costs, difficulty measuring multiple persons at the same time, and the requirement of expert knowledge. These limitations restrict the use of optoelectronic-based motion analysis to research settings. The use of inertial measurement units (IMUs) overcomes these issues and allows for human motion analysis outside laboratory settings [2]. An IMU consists of a combination of accelerometers, gyroscopes, and optionally magnetometers. Measurements from these sensors are combined in a process called ‘sensor fusion’ to obtain the orientation of the IMU. Therefore, when IMUs are fixated to body segments, their orientation in space can be estimated. Consequently, recent years have seen an increased interest in IMUs in sports specific settings.

Multiple studies have validated IMUs with optoelectronics for whole body kinematics [3], lower body kinematics during gait [4,5], lower body kinematics during dynamic movements [6,7], and upper body kinematics [8,9]. Relative joint angle errors between both methods were present in all studies and ranged from 2 to 14 degrees root-mean-square error (RMSE). With IMU-based motion analysis, these joint angles are determined by calculating the relative orientation between IMUs on adjacent body segments. Therefore, errors in the orientation estimation of both body segments are propagated to the joint angle errors. To the best of our knowledge, no study has quantified the errors of body segment orientations separately. Moreover, no study has separated these errors to the different sources of error that exist in IMU-based human motion analysis. There are three important sources of error to consider: the definition of the body frames, the soft tissue artefact (STA), and the orientation filter.

Body frames are defined directly from 3D marker positions when using optoelectronics. IMUs, however, have their own local coordinate frame attached to the sensor, necessitating a sensor-body calibration procedure to define body frames. These procedures incorporate pre-defined postures [10,11,12] or movements [13,14], which must be executed by the person being measured. This means that the accuracy of the sensor-body calibration depends on the execution of the calibration movements or postures. Consequently, the definitions of the body frames may differ between optoelectronics and IMU-based motion analysis methods.

The Soft Tissue Artefact (STA) is defined as the error caused by the relative motion between the bone and the skin. STA is possibly the biggest source of error in the field of human motion analysis [15]. Both optoelectronics and IMUs suffer from STA. However, optoelectronic systems are less vulnerable to STA because it measures marker positions. Markers are relatively far apart, and marker locations are chosen such that relative motion between the markers and bone is minimized. Therefore, the effects on the modeled body segment orientations may be relatively small. An IMU, on the other hand, is directly affected by a change in orientation of the skin with respect to the bone. Especially in sports, this may be a problem because of muscles bulging when contracting, and because of inertia effects around moments of impact with the ground. However, since STA quantification is difficult and has so far been limited to small scale, low activity studies [16], no studies have quantified the STA of IMUs.

To estimate the orientation of an IMU, the measurements of the rate gyroscopes are integrated. However, due to integration drift, the computed orientation would suffer from large errors within seconds. To compensate for this drift, both the accelerometers and magnetometers are assumed to measure a constant vector, gravity, and the earths’ magnetic field, respectively. An orientation filter combines orientation estimates based on these vectors with the orientation estimates based on the rate gyroscope measurements to obtain a better sensor orientation estimate [17]. In the case of magnetic field distortions or linear accelerations, the assumption of a constant vectors no longer holds true, which leads to orientation errors [18].

To the best of our knowledge, no study has been performed separating the error of IMU-based body segment orientations in the definition of the body frames, the STA, and the orientation filter. Consequently, methods to minimize the various errors are currently being used without knowing their exact influence. Therefore, the goal of this study is to present a method to quantify the sources of body segment orientation errors in human motion analysis with IMUs. The errors are quantified for the example of football movements, which we think would be representative for other field sports as well. In addition, various calibration techniques, movement types, and movement intensities are compared to examine their influence on the different sources of error. The quantification of these errors can provide a necessary insight for all human motion analysis studies using IMUs and would allow for the optimization of error reduction techniques. In addition, raw data and accompanying software to quantify the different sources of error are openly available to allow other researchers to optimize their own data processing methods, and to quantify the performance of different data processing methods. It was hypothesized that different calibration movements, movement intensities, movement types, and different body segments, would influence the different sources of error of human motion analysis with IMUs.

## 2. Materials and Methods

### 2.1. Subjects and Instrumentation

**Figure 1:**
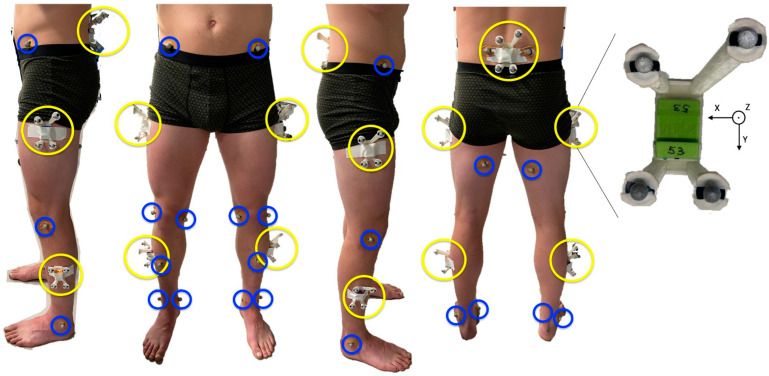
Subject instrumentation. IMUs were placed in the Rigid Marker Clusters and placed on the left and right thigh, left and right shank and pelvis, represented by yellow circles in the figure. An enlarged RMC is shown on the right-hand side. Markers were placed on the following anatomical landmarks: the anterior and posterior superior iliac spines, the posterior side of the thighs halfway the length hip to knee, the medial and lateral femoral epicondyles, the anterior side of each shank halfway the length knee to ankle and the medial and lateral malleoli, represented by blue circles in the figure.

Thirteen male subjects (mean ± std: age [y] 22.4 ± 2.2, height [cm] 182 ± 8.3, weight [kg] 72.2 ± 10) participated in the study. However, due to technical issues, the data of three participants could not be used for data analysis. All subjects were active football players with at least five years of experience and had their right leg as their preferred leg of shooting. All subjects signed an informed consent in accordance with the Declaration of Helsinki and the study was approved by the ethics committee of the VU Amsterdam. No subjects had any injury at the time of testing. Subjects were equipped with five IMUs (MPU-9150, Invensense, SanJose, CA, USA). The IMUs were placed on the left and right thigh, left and right shank and on the pelvis, see Figure 1. Data was gathered at 500 Hz and logged on a SD card inside the IMU, allowing for offline analysis.

Sixteen retro-reflective markers were placed on the following anatomical landmarks: the anterior and posterior superior iliac spines, the posterior side of the thighs halfway the length hip to knee, the medial and lateral femoral epicondyles, the anterior side of each shank halfway the length knee to ankle and the medial and lateral malleoli, see Figure 1. The location of the markers was captured at 250 Hz with cameras of the Vicon Optoelectronic system (Eight Vicon V5 cameras, Vicon Motion Systems Ltd., Oxford, UK).

A Rigid Marker Cluster (RMC) is a rigid connection between 4 markers and the IMU [6]. A 3D model was designed in SolidWorks (SolidWorks 2015, Dassault Systèmes, Winchester, MA, USA) and printed in PLA with an Ultimaker 3D printer, weighing 41 g including IMU, see Figure 1. The position of these markers was also captured at 250 HZ with the Vicon Optoelectronic system. The local coordinate system of the RMCs was defined so that when an IMU was placed inside the RMC, their coordinate systems were aligned.

### 2.2. Protocol

The IMUs were turned on and time synchronized with a mechanical peak in the accelerometers [19]. The IMUs and markers were fixed to the RMCs with double sided adhesive tape. The RMCs and markers were placed on shaved skin with double sided adhesive tape, see Figure 1.

**Figure 2:**
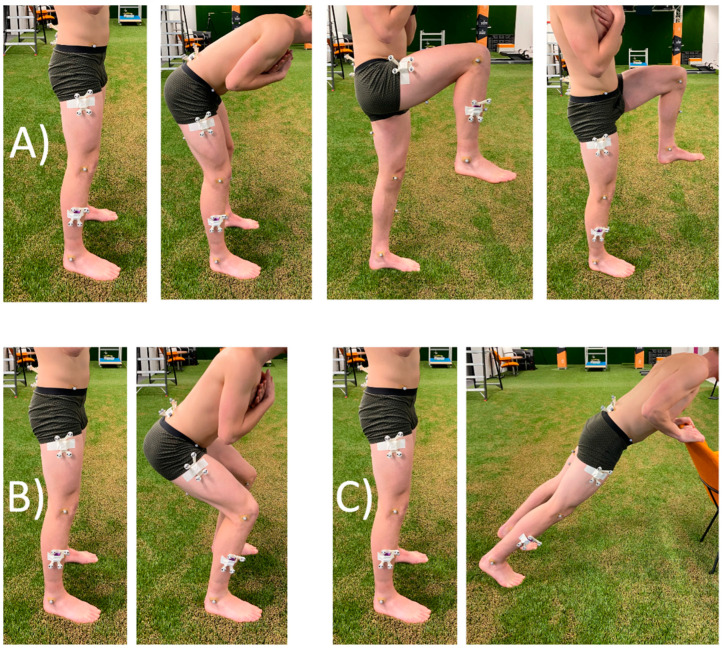
Sensor-body calibration movements. Calibration (**A**) consists of a neutral pose followed by a bow, right thigh rise and left thigh rise. Calibration (**B**) consists of a neutral pose and a squat movement. Calibration (**C**) consists of a neutral pose and an inclined plank movement.

A sensor-body calibration was necessary to construct coordinate systems for each body segment. Three different sensor-body calibrations were performed. All calibration methods had the same first step, a static calibration in the neutral pose. In this position the longitudinal axis of each body segment was assumed to be parallel to gravity and could therefore be defined. The second step of the sensor-body calibration was a movement performed in the sagittal plane and could therefore be used to define the frontal axis of the body segments. The third axis of the coordinate systems of the body segments was defined orthogonal to the longitudinal and horizontal axis [7]. The three different sensor-calibration movements were: bow and thigh rise (A), squat (B) and inclined plank (C) [12], see Figure 2. For the inclined plank movement, the subject had to place his hands on an object in front him and move towards this object while keeping his legs and torso in a straight line.

After the sensor-body calibrations, three types of movement were executed at three intensities. The nine trials were: a squat, a squat jump at 50% intensity, a squat jump at 100% intensity, a walk, a run at 50% intensity, a sprint at 100% intensity, a pass, a cross and a shot. Because the criterion to control movement intensity were qualitative, there may have been some variability in movement execution within and between subjects. However, if a higher movement intensity would result in larger errors, this would still show in the results. All subjects used their right leg as their kicking leg. Each trial was repeated eight times.

### 2.3. Data Processing

The raw marker position data was processed in the Vicon Nexus (version 2.7.3, Vicon Motion Systems Ltd., Oxford, UK). Possible gaps in marker trajectories were filled using Nexus’ Woltring gap fill algorithm, the rigid body gap fill and the kinematic gap fill for gaps of a maximum of 0.05, 0.1 and 0.7 s, respectively, according to the recommendations by Visual 3D (C-Motion, Inc, Germantown, MD, USA). Of the eight recorded trials, the first five trials without gaps larger than 0.7 s were used for further processing. Marker data was exported to Matlab (version 2020a, The MathWorks, Inc., Natick, MA, USA). Based on the recommendations by Visual 3D (C-Motion, Inc, Germantown, MD, USA) to reduce marker position noise, a fourth order low-pass Butterworth filter with a cut-off frequency of 8 Hz was applied to all marker trajectories. Based on the markers on the anatomical landmarks, in combination with a measurement of the leg length, body frames were defined for the left and right thigh, left and right shank and the pelvis, based on Cappozzo et al. [20] and following ISB recommendations [21]. These body frames were named the Anatomical Landmark marker calibrated body frames (AL-mcbf). The markers on the RMC were used to construct an RMC sensor frame (RMC-sf), which was aligned with the IMU sensor frame (IMU-sf). Both the orientation of the AL-mcbf and the RMC-sf frames were described as rotation quaternions relative to the global reference frame (GRF) of the Vicon system. Rotation quaternions were used since they provide a continuous description of orientation and do not suffer from difficulties such as gimbal lock.

Offsets and misalignment in the IMU sensors were compensated for with an IMU-sensor calibration [22,23]. A gradient descent Madgwick algorithm with a filter gain of 0.043 [17] was used to compute the orientation of the IMU sensor frame (IMU-sf) relative to the IMU-Reference frame (IRF). Thereafter, IMU was down sampled to 250 Hz to match the sample frequency of the marker data. Furthermore, the IMU data was synchronized with the marker data according to [7]. This means that besides a potential synchronization error, the maximal remaining phase error was 0.002 s. The already described sensor-body calibration movements were used to compute the IMU-body frames. The sensor-body calibration procedure was based on Wilmes et al. [7] and followed the ISB recommendations [21]. Body frames were constructed for each of the different sensor-body calibration movements. These frames were named the IMU sensor calibrated body frames A–C (IMU-scbf(A–C)) based on which calibration movement was used, see Equation (1), qAB is the rotation quaternion from frame A to frame B, $q_{t}$ is a time varying rotation quaternion and q is a time invariant rotation quaternion).

(1) qtIRFIMU−scbf(A−C)=qtIRFIMU−sf ⊗ qIMU−sfIMU−scbf(A−C)

To compare the quaternions based on the IMU measurements with the quaternions based on the optoelectronic measurements, they must be described with respect to the same global reference frame using a hand-eye calibration [24]. A large compass with markers at both ends was used to determine the quaternion between the IRF and GRF [17]. The compass was placed in the middle of the measurement volume and thereby as far away from any electrical wiring as possible to minimize the potential influence of local magnetic field distortions on the hand-eye calibration. The hand-eye calibration quaternion was then used to describe the IMU-sf and the IMU-scbf(A–C) relative to the GRF, see Equations (2) and (3):

(2) qtGRFIMU−sf=qGRFIRF ⊗ qtIRFIMU−sf

(3) qtGRFIMU−scbf(A−C)=qGRFIRF ⊗ qtIRFIMU−scbf(A−C)

**Figure 3:**
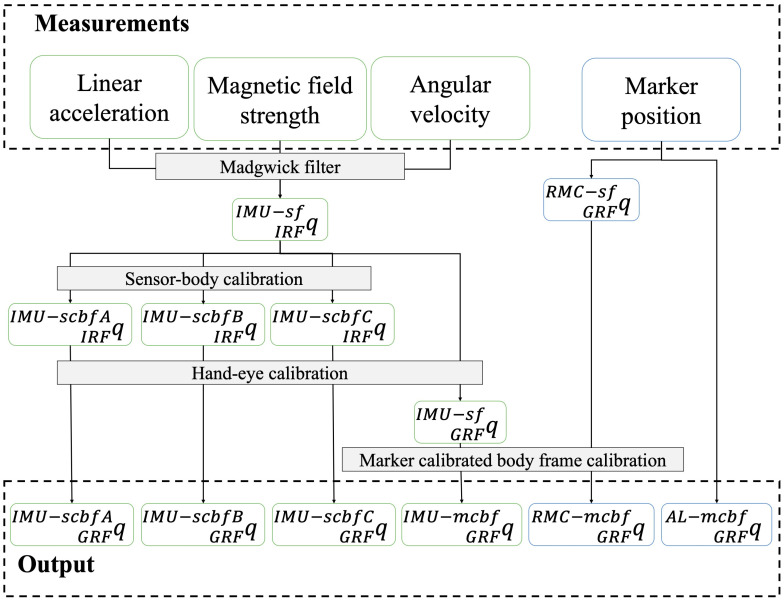
Data processing flow chart. Measurements were converted to rotation quaternions between two frames. IRF = IMU Reference Frame, GRF = Global Reference Frame, IMU-sf = IMU sensor frame, IMU-scbf(A–C) = IMU sensor calibrated body frame A, B and C, RMC-sf = RMC sensor frame, AL-mcbf = Anatomical Landmarks marker calibrated body frame, IMU-mcbf = IMU marker calibrated body frame, and RMC-mcbf = RMC marker calibrated body frame. The sensor-body calibration is based on the different calibration movements(A–C). The hand-eye calibration is based on the marker compass measurement. The marker calibrated body frame calibration is based on a static trial.

Furthermore, to quantify the error of the definition of the body frames, the IMU-sf and RMC-sf were rotated to a marker calibrated body frame, based on the static trial, see Equations (4)–(7). An overview of all data processing steps is provided in Figure 3.

(4) qAL−mcbfIMU−sf=1n∑t=1n((qtGRFAL−mcbf)−1 ⊗ qGRFIMU−sft)

(5) qtGRFIMU−mcbf=qtGRFIMU−sf ⊗ qIMU−sfAL−mcbf

(6) qAL−mcbfRMC−sf=1n∑t=1n((qtGRFAL−mcbf)−1 ⊗ qtGRFRMC−sf)

(7) qtGRFRMC−mcbf=qtGRFRMC−sf ⊗ qRMC−sfAL−mcbf

### 2.4. Data Analysis

After data processing, six different body frames (IMU-scbfA, IMU-scbfB, IMU-scbfC, IMU-mcbf, RMC-mcbf, and AL-mcbf) are described relative to the global frame for each body segment. Table 1 shows the comparisons between the frames that were made.

As described in the introduction, there were three main sources of error between IMUs and an optoelectronic system: the definition of the body frames, the soft tissue artefact (STA), and the orientation filter. In comparison #1–3 the same measurements were used to compute the orientation of the body segments. However, since different sensor-body calibration methods were used, the difference in these comparisons is the error caused by the definition of the body frames. In comparison #4 the frames were constructed from measurements of the optoelectronic system and were therefore not influenced by the orientation filter. In addition, the frames were calibrated to the same body frame. Consequently, comparison #4 entails the relative motion between the body frames obtained using the markers on bony landmarks and the body frames obtained using the rigid marker cluster with a rigid connection to the IMU. This relative motion is caused by the STA. In comparison #5, there was a rigid connection between the IMU and the RMC preventing the influence of STA. Additionally, because both frames were aligned, no error from the definition of the body frames was present. Therefore, the error found in comparison #5 was the error caused by the orientation filter. In comparison #6 the orientations obtained from the IMUs were expressed in the same body frame as the orientations obtained from the markers on bony landmarks, ruling out the error of body frame definitions. Therefore, the difference was the combined error of the orientation filter and the STA. In comparison #7–9 the IMU body frames were calibrated based on the calibration movements (A–C). Therefore, the difference in these comparisons included the error caused by the definition of the local frames, the STA, and the orientation filter.

Each comparison was described by the rotation quaternion between the two frames. From this quaternion the smallest rotation between the two frames was calculated in degrees. For each trial the root mean square difference (RMSD) of the smallest angle between the frames was calculated. For each subject the mean over five repetitions of all nine different trials was calculated. Subsequently, the mean and standard deviation (STD) of the RMSD over all subjects was calculated for each trial and each body segment. A three-way repeated measures ANOVA with Bonferroni post-hoc comparisons were performed to assess the effects of movement type, intensity, and body segment (3 movement types x 3 intensities x 5 segments) on the STA errors, orientation filter errors, the total errors. A Greenhouse-Geisser correction was applied if the assumption of sphericity was not met. The significance level was set at *p* < 0.05 and effect size was calculated as eta squared (η^2^).

## 3. Results

### 3.1. Definition Body Frames

**Figure 4:**
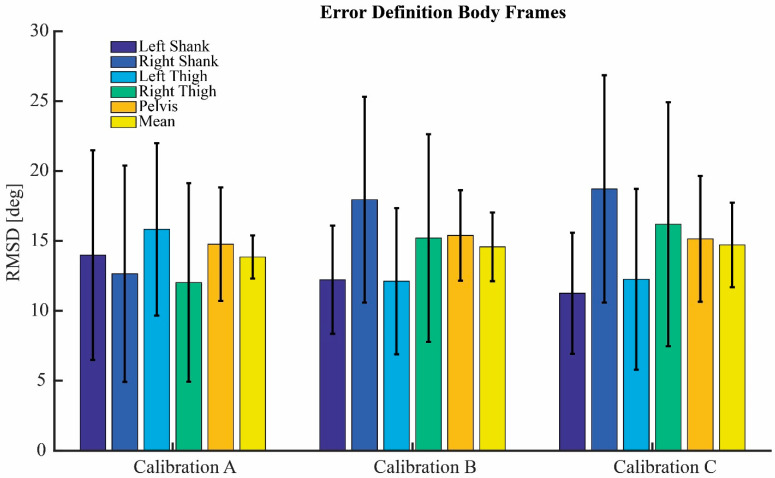
Error of the definition of the body frames. Root-mean-square differences (RMSD) of all body segments for the three different calibrations, see Figure 3 for calibration movements.

Figure 4 shows the RMSD and STD of the error of the definition of the body frames for calibration A, B, and C. The error of the definition of the body frames was constant over time and thus constant for all trials of each subject. No calibration method significantly outperformed the others. The errors ranged between 11.3 and 18.7 degrees RMSD. All three calibration methods showed large between subject variability with a STD of up to 8.8 degrees.

### 3.2. Soft Tissue Artefact

**Figure 5:**
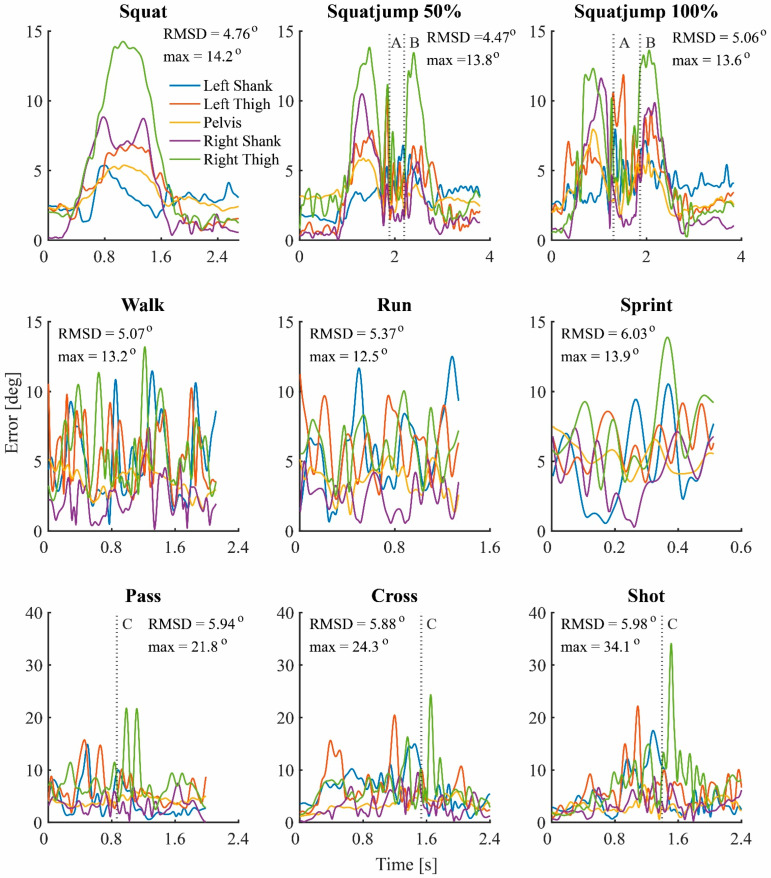
Error caused by the soft tissue artefact, plotted for each trial over time of a typical subject. In the top right corner of each plot the root-mean-square difference (RMSD) across all body segments and maximum error are displayed in degrees. The vertical dashed line represents a specific moment in time: A = push off, B = landing, C = ball contact.

The repeated measures ANOVA results of the effects on the errors caused by STA are shown in Table 2. Movement type, intensity, and segment were all significant main effects. The results of all Bonferroni post-hoc tests and the results of a single subject were included in the Appendix A. The RMSD of the STA ranged from 3.8 degrees on the pelvis during the walk trial, to 9.1 degrees on the right thigh during the sprint trial. Figure 5 shows the error caused by the STA plotted over time for each trial of a random subject. In the top right corner of each plot, the mean RMSD overall body segments and the maximum error are displayed in degrees. It can be seen that the STA is not a constant offset or white noise, but that the error is very much time dependent. For the squatting trials, the STA arises during the flexion of the knees, both before push-off and after landing for the squat jumps. For the walk, run, and sprint trial, the STA shows a cyclic pattern related to the gait cycle. The shooting trials display a spike of the left thigh before ball contact and a spike of the right thigh directly after ball contact. The spike before ball contact is caused by the landing of the stance leg, the spike after ball contact by the ball contact and the swing through of the shooting leg. The maximum error in time occurred during the shot trial on the right thigh and was 34.4 degree.

### 3.3. Orientation Filter

**Figure 6:**
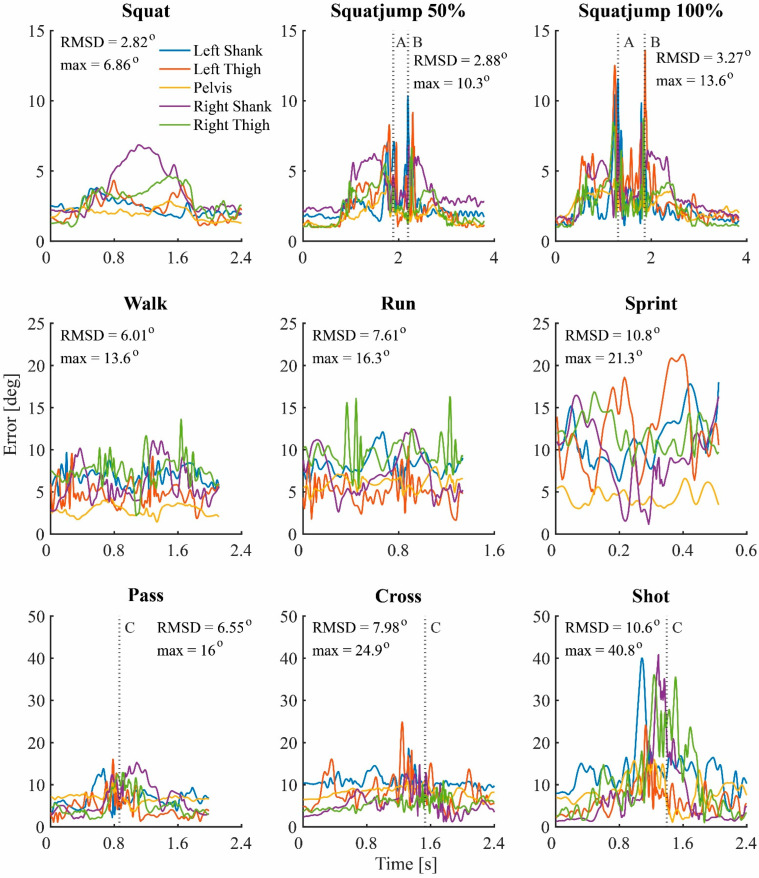
Error of the orientation filter, plotted for each trial over time of a typical subject. The exact same trails were used as in Figure 5. In the top right corner of each plot the root-mean-square difference (RMSD) across all body segments and maximum error are displayed in degrees. The vertical dashed line represents a specific moment in time: A = push off, B = landing, C = ball contact.

The repeated measures ANOVA results of the effects on the errors caused by the orientation filter are shown in Table 2. Movement type, intensity, and segment were all significant main effects. The RMSD of the error caused by the orientation filter ranged from 3.0 degrees on the left shank during the squat trial to 12.7 degrees on the left shank during the sprint trial. Figure 6 shows the error caused by the orientation filter plotted over time for the same subject and the same trials as in Figure 5. The error caused by the orientation filter is highly time dependent. The error over time consists of a constant offset of around 1 degree, some high frequency behavior around moments of impact and some low frequency behavior related to movement. For the squatting trials, the error of the orientation filter arises during the flexion of the knees, both before push-off and after landing for the squat jumps. For the walk, run, and sprint trial, the error of the orientation filter shows a cyclic pattern related to the gait cycle. The shooting trials display high frequency behavior around the moment of ball contact. A relationship between movement intensity and maximum error in time is visible. The maximum error in time occurred during the shot trial on the right shank and was 41.2 degrees.

### 3.4. Total Error

**Figure 7:**
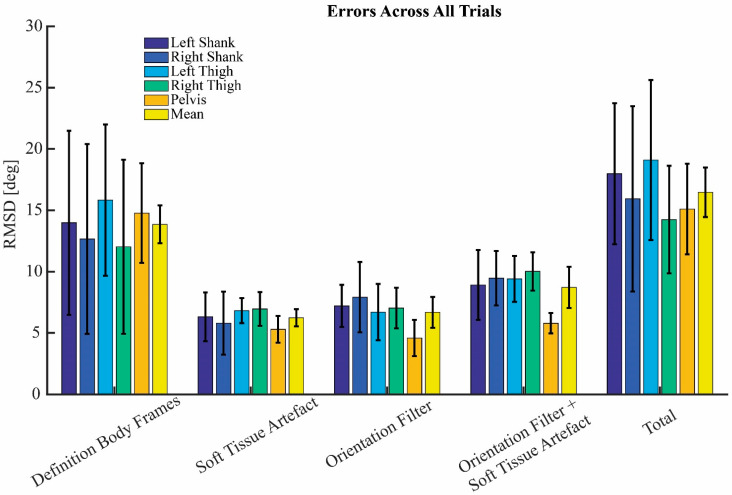
Root-mean square differences (RMSD) and standard deviations for each of the sources of error, and total error, for all body segments, mean over all trials. The error of the definition of the body frames and the total error is displayed for calibration A.

Figure 7 shows the mean RMSD and STD for each of the sources of error, for the combined error of the STA and the orientation filter, and for the total error, averaged over all trials. To reduce the amount of displayed data, the error of the definition of the body frames and the total error is only displayed for sensor-body calibration A. The mean RMSD and STD of the combined error of the STA and the orientation filter and of the total error are also shown for each trial in the Appendix A.

## 4. Discussion

The goal of this study was to present a method to quantify the sources of body segment orientation errors in human motion analysis with IMUs. In addition, the influence of different sensor-body calibration methods, different movements, and movement intensities on the sources of error were studied. The sources of error were quantified by comparing the IMUs against an optoelectronic system. It should be noted that the optoelectronic system is not a gold standard in all cases and therefore the results were described by root-mean-square differences (RMSD) instead of root-mean-square error. Another important notion is that because the goal of this study was to get a complete insight in the different sources of error, no error minimization techniques were used on the IMU data. In current literature this is not the case. Furthermore, unlike current literature, this study described the orientation of body segments instead of joint angles. This choice was also made to get a greater insight in the different sources of error, since joint angle errors comprise the errors of the IMU on the body segment proximal to the joint and the errors of the IMU on the body segment distal to the joint.

### 4.1. Definition Body Frames

The definition of the body frames was the largest source of error for the IMUs. The high between subject variability shows that there are subject specific factors that cause a difference. This difference could arbitrarily be attributed to either the IMUs or the optoelectronic system, depending on which one is chosen as the gold standard. The high between subject variability could be caused by the inconsistent execution of the calibration movements, or by inconsistent anthropometry or marker placement, which would influence the marker calibrated body frames.

A study by Robert-Lachaine et al. [3] also compared the defined body frames of IMUs with an optoelectonic system. The results ranged from 2.5 to 7.0 degrees RMSE of the joint Euler angles of the lower extremities. This considerably smaller error could be explained by the fact that this study used the Xsens MVN model. The MVN model uses a combination of static postures, anthropometric measures, and boundary conditions to define body frames [25].

The used method in this study was able to quantify the error of the definition of the body frames. The definition of the body frames with IMUs could possibly be improved by using models, such as the Xsens MVN model. However, the great advantage of IMUs is their ease of use, easy placement method, and short set-up time. Therefore, there exists a trade-off between the accuracy of the definition of the body frames and practicality for each specific application. However, as shown in current study, differences in the method to define body frames can lead to relatively large differences in estimated body segment orientations. A standardized method for body frame definitions using IMUs would allow for better comparison between studies.

### 4.2. Soft Tissue Artefact

High frequency STA was seen at moments of impact, such as the landing of the squat jumps and around the moment of ball contact for the shooting trials. However, especially for the squat trials, the range of motion had a much larger influence on the RMSD of the STA. This part of the STA is caused by skin sliding and is related to the flexion or extension of a joint close to the IMU. This can be seen in Figure 5 by the high STA of the pelvis during the squat trials, during which there is lower back flexion. In all other trails there is little or no lower back flexion, and thus less STA on the pelvis.

A limitation of the current method is that the STA is quantified relative to an optoelectronic system, of which the markers also suffer from STA. Gold standard STA quantification techniques require either the surgical placement of bone pins [26] or the use of medical imaging techniques, such as fluoroscopy [27]. However, these techniques are highly unsuitable for dynamic activities. Consequently, there is no available literature regarding STA in dynamic sports activities to compare present results to. Since the markers of the optoelectronic system are placed on bony landmarks, and the IMUs somewhere on the middle of a segment, the relative STA between the two systems was assumed to be a good approximation of the absolute STA of the IMUs. Another limitation of the current method is that the IMUs were placed in the RMCs. This increases the mass of the object fixated on the skin, and because part of the STA is caused by inertia, the STA is likely to be overestimated in this study.

**Figure A5:**
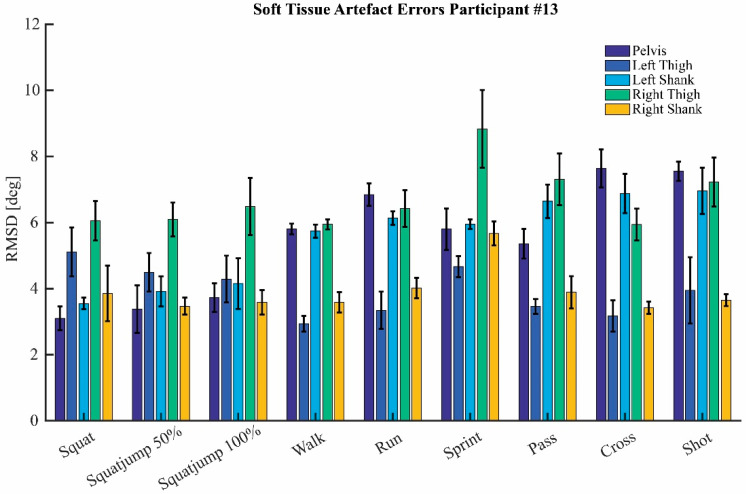
Error caused by the soft tissue artefact of a typical subject. Mean root-mean-square differences (RMSD) and standard deviations for all body segments and all trials.

To the best of our knowledge, the current study is the first study to quantify the STA during dynamic activities and the first study to quantify the STA of IMUs. The used method was able to show the dependence of STA on movement type, movement intensity, and body segment. However, the noted limitations should be taken into consideration when interpreting the absolute values of the results. Yet, the low within subject variability (see Appendix A, Figure A5) does indicate good repeatability of the method.

### 4.3. Orientation Filter

**Figure A6:**
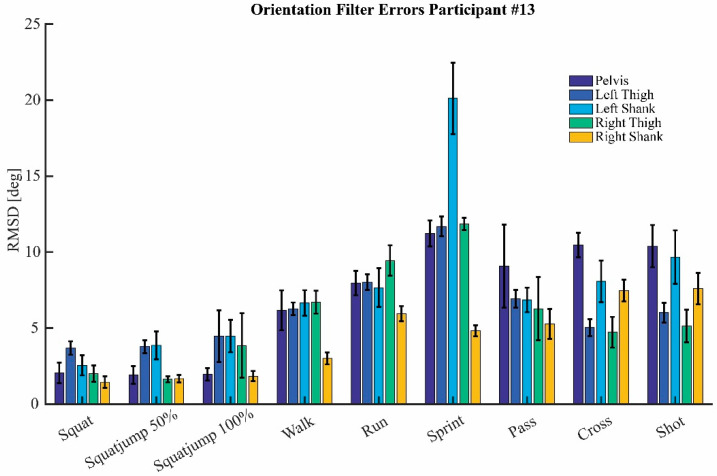
Error caused by the orientation filter of a typical subject. Mean root-mean-square differences (RMSD) and standard deviations for all body segments and all trials.

Although there was high variability in the results, a dependency of the error of the orientation filter on movement type, intensity, and segment was shown. However, the within subject variability was much lower (the results of a single subject are displayed in the Appendix A, see Figure A6). This indicates that the high variability is caused by between subject factors. The main factor is expected to be the use of different IMUs. Although all IMUs were both initialized and calibrated in the same way, a difference in performance was noticed. In Figure 6, the error caused by the orientation filter could be split up in a constant offset, low frequency behavior, and high frequency behavior. The constant offset and the low frequency behavior could have different causes, including the misalignment of the sensors within the IMU or noise in sensor measurements. Furthermore, despite careful sensor calibration procedures, small biases in the sensor measurements may still have been present or may have reappeared after calibration. The high frequency behavior is expected to be caused by moments of impact, such as landing after a squat jump or around the moment of ball contact. During these moments, the body segments undergo substantial accelerations, making the direction of the resultant acceleration measured by the accelerometers no longer aligned with gravity. In addition, potential distortions in the local magnetic field could have contributed to orientation filter errors because the assumption that the magnetometer measures the earth’s magnetic field only is no longer met.

A study by Cavallo et al. [28] used a robotic arm to quantify the error of different orientation filters. For slow rotations (18 degrees/s) the Madgwick filter had an error of 5.13 degrees RMSE and 7.07 degrees RMSE for fast rotations (45 degrees/s). Since, in this study, the IMU was only rotated, the influence of linear accelerations was not captured. Furthermore, during the sprint trials, angular velocities of up to 1000 degrees/s were measured, which are of a far larger magnitude than the study by Cavallo et al. [28]. Another difference with the current study is that a robotic arm was used as gold standard instead of an optoelectronic system. At dynamic moments of impact and high accelerations, there were gaps in the marker data, which were filled using gap fill algorithms. Especially for dynamic movements, the use of these gap fill algorithms, but also the use of low pass filters, could lead to a reduction of the actual peak orientations. However, based on the low within subject variability (see Appendix A, Figure A6) and the agreement in magnitude with previous studies, we are confident that with present method we were be able to quantify the error caused by the orientation filter.

### 4.4. Total Error

The total error captured the combined effect of each of the three individual errors. Additionally, the combined effect of the STA and the error caused by the orientation filter was quantified. The combined effect was approximately the square root of the square of the individual errors, for both the combined effect of the STA and orientation filter, and for the total error. This implies that the individual errors are roughly independent of one another.

A study by Wilmes et al. [7] validated IMUs with an optoelectronic system for a range of dynamic football movements. Across all movements, an average error of 5.3 degrees RMSD was reported for knee flexion/extension angles and 8.0 degrees RMSD for hip flexion/extension angles. These lower RMSDs are expected to be caused by the use of filter techniques on the IMU data. In the current study, no filtering techniques were used to get insight in the complete error caused by the different sources. Another difference is the use of joint angles instead of segment orientations.

## 5. Conclusions

This study showed a method to quantify the different sources of body segment orientation errors of human motion analysis with IMUs. During dynamic football activities, the errors of definition of the body frames ranged from 11.3 to 18.7 degrees RMSD, the STA errors ranged from 3.8 to 9.1 degrees RMSD, and the error caused by the orientation filter ranged from 3.0 to 12.7 degrees RMSD. The type of movement, movement intensity, and body segment, were found to have a significant effect on the errors. Future studies should take these errors into account since they influence IMU-based human motion analysis. In addition, this study highlights the importance of standardizing the methods to define body frames with IMUs. Furthermore, both raw data and the data processing scripts of the current study were openly published to give other researchers the opportunity to optimize their own data processing methods, and to quantify the performance of different data processing methods.

## Figures and Tables

**Table 1:** Comparisons between the different body frames and the error that the difference between frames describes.

Comparisons	Frame 1	Frame 2	Error
#1	IMU-scbfA	IMU-mcbf	Error definition body frames A
#2	IMU-scbfB	IMU-mcbf	Error definition body frames B
#3	IMU-scbfC	IMU-mcbf	Error definition body frames C
#4	RMC-mcbf	AL-mcbf	Soft Tissue Artefact
#5	IMU-mcbf	RMC-mcbf	Error orientation filter
#6	IMU-mcbf	AL-mcbf	Error orientation filter + Soft Tissue Artefact
#7	IMU-scbfA	AL-mcbf	Total error with calibration A
#8	IMU-scbfB	AL-mcbf	Total error with calibration B
#9	IMU-scbfC	AL-mcbf	Total error with calibration C

**Table 2:** Significance and effect size of main and interaction effect of movement type, intensity, and segment on the root-mean-square difference of the error caused by the soft tissue artefact (STA), the orientation filter, and the total error. Insignificant effects were denoted with an asterisk (*).

	Error STA		Error Orientation Filter		Total Error	
Effects:	*p*	η^2^	*p*	η^2^	*p*	η^2^
Movement type	<0.001	0.100	<0.001	0.201	<0.001	0.046
Intensity	<0.001	0.052	<0.001	0.066	<0.001	0.008
Segment	<0.001	0.154	0.018	0.104	0.604 *	0.040
Movement type: Intensity	<0.001	0.042	<0.001	0.063	0.046	0.003
Movement type: Segment	<0.001	0.132	0.006	0.055	<0.001	0.038
Intensity: Segment	0.750 *	0.004	<0.001	0.019	<0.001	0.008
Movement type: Intensity: Segment	0.001	0.025	0.003	0.012	<0.001	0.009

**Figure A1:**
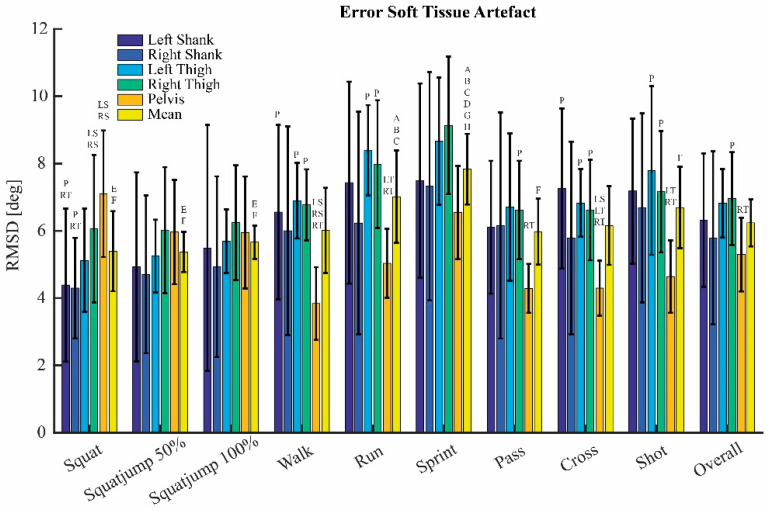
Error caused by the soft tissue artefact. Mean root-mean-square differences (RMSD) and standard deviations for all body segments and all trials. Significant differences within each trial between body segments are displayed by letters above the bar, LS, RS, LT, RT, and P mean a significant difference with the left shank, right shank, left thigh, right thigh, and pelvis, respectively. Significant differences between trails are displayed with a letter above the mean bar. A = squat, B = squat jump 50%, C = squat jump 100%, D = walk, E = run, F = sprint, G = pass, H = cross and I = shot.

**Figure A2:**
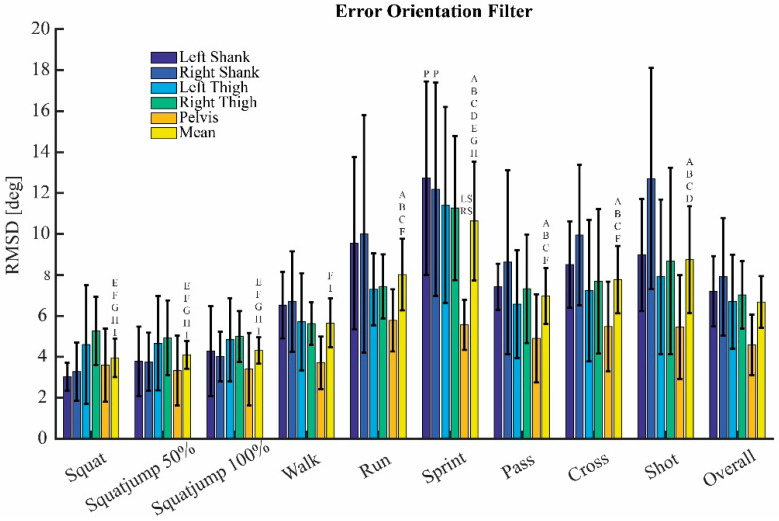
Error of the orientation filter. Mean root-mean-square differences (RMSD) and standard deviations for all body segments and all trials. Significant differences within each trial between body segments are displayed by letters above the bar, LS, RS, LT, RT, and P mean a significant difference with the left shank, right shank, left thigh, right thigh, and pelvis, respectively. Significant differences between trails are displayed with a letter above the mean bar. A = squat, B = squat jump 50%, C = squat jump 100%, D = walk, E = run, F = sprint, G = pass, H = cross and I = shot.

**Figure A3:**
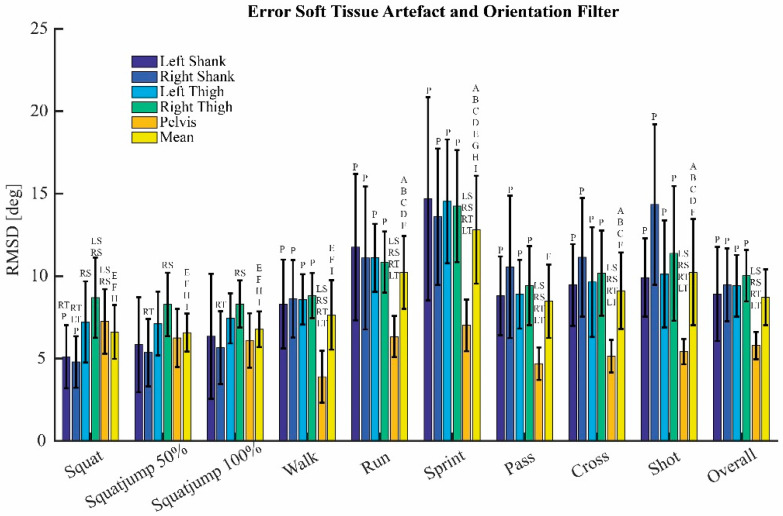
Combined error of the soft tissue artefact and the orientation filter. Mean root-mean-square differences (RMSD) and standard deviations for all body segments and all trials. Significant differences within each trial between body segments are displayed by letters above the bar, LS, RS, LT, RT, and P mean a significant difference with the left shank, right shank, left thigh, right thigh, and pelvis, respectively. Significant differences between trails are displayed with a letter above the mean bar. A = squat, B = squat jump 50%, C = squat jump 100%, D = walk, E = run, F = sprint, G = pass, H = cross and I = shot.

**Figure A4:**
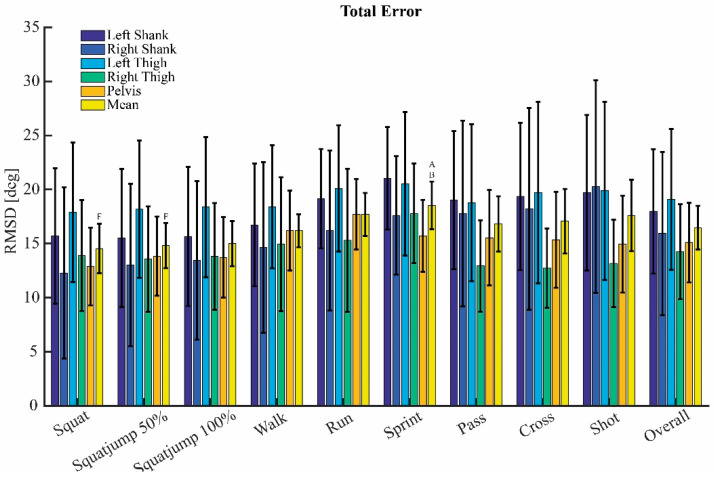
Total error with sensor-body calibration A. Mean root-mean-square differences (RMSD) and standard deviations for all body segments and all trials. Significant differences within each trial between body segments are displayed by letters above the bar, LS, RS, LT, RT, and P mean a significant difference with the left shank, right shank, left thigh, right thigh, and pelvis, respectively. Significant differences between trails are displayed with a letter above the mean bar. A = squat, B = squat jump 50%, C = squat jump 100%, D = walk, E = run, F = sprint, G = pass, H = cross and I = shot.

## Data Availability

The data and Matlab scripts for the data processing steps that support the findings of this study are openly available in Zenedo at http://doi.org/10.5281/zenodo.6610795 (accessed on 9 December 2022).
